# Reproductive aging, preimplantation genetic testing for aneuploidy, and the diameter of blastocysts: does size matter?

**DOI:** 10.18632/aging.206215

**Published:** 2025-03-05

**Authors:** Jakub Wyroba, Joanna Kochan, Maria Barszcz, Grzegorz Mirocki, Pawel Kordowitzki

**Affiliations:** 1 Malopolski Institute of Fertility Diagnostics and Treatment – KrakOvi, Krakow, Poland; 2 Fertility Disorders Clinic, Andrzej Frycz Modrzewski Krakow University, Krakow, Poland; 3 Department of Animal Reproduction, Anatomy and Genomics, University of Agriculture in Krakow, Krakow, Poland; 4 Department of Cell Biology, Harvard Medical School, Boston, MA 02115, USA; 5 Department of Preclinical and Basic Sciences, Nicolaus Copernicus University, Torun, Poland; 6 Department of Gynecology Including Center of Oncological Surgery (CVK), Charité, Berlin, Germany

**Keywords:** maternal age, embryo, blastocyst, preimplantation genetic testing, aneuploidy

## Abstract

There is no doubt that maternal aging, also known as reproductive aging, can contribute to the increased rates of aneuploidy observed in blastocysts generated from women of advanced age who undergo *in vitro* fertilization (IVF). Additionally, the hatching process of the blastocyst, which is crucial for successful implantation, may be impaired in aneuploid embryos. Aneuploid embryos often exhibit abnormal cell division and chromosomal distribution, which can lead to disruptions in the hatching process. Due to ethical restrictions, preimplantation genetic testing for aneuploidy (PGT-A) is unavailable in all countries. Therefore, our retrospective study of 502 couples who underwent intracytoplasmic sperm injection (ICSI) aimed to elucidate if embryonic features, such as the ability to hatch and embryonic diameter, could be a reliable estimator for the success rate after embryo transfer, especially for women aged 26–45 years, and for IVF clinics which do not have access to PGT-A. The small hatching blastocysts (Bl. 5) group had a significant (*p* < 0.001) higher percentage of euploid embryos (≤35 Y- 73%, >35Y- 51%) compared to large (Bl. 4) counterparts (≤35 Y-58%, >35 Y- 38%). In patients aged 34-38 years, we detected 10% more euploid blastocysts in the hatching group than the expanding ones, which was a significant difference (*p* < 0.05). In conclusion, when selecting non-PGT-A tested embryos for embryo transfer (ET) or frozen embryo transfer (FET), a small hatching blastocyst seems to be a better choice than a large expanded one, especially for advanced-age patients for whom the risk of aneuploidy is higher.

## INTRODUCTION

Preimplantation genetic testing for aneuploidy (PGT-A) has emerged as a powerful tool in assisted reproductive technology, offering the potential to enhance the chances of successful embryo implantation and pregnancy. The detection of chromosomal abnormalities, or aneuploidies, in preimplantation embryos has long been a concern in reproductive medicine. Effective noninvasive screening tests for aneuploidies have been developed and are superior to maternal age alone as a method of identifying candidates for invasive testing [1]. As the global population continues to age, the issue of maternal aging and its implications on *in vitro* fertilization has become a topic of increasing concern and scholarly interest [2]. Women are now delaying childbearing, with the average age of first birth rising from 21 in 1970 to 26.9 in 2018 [3]. This trend has been driven by social and cultural advancements, as well as significant progress in artificial reproductive technology, which has enabled women of advanced maternal age to conceive [4]. However, this shift has also brought to light the challenges associated with advancing maternal age. After the age of 35, rates of infertility, chromosomal abnormalities, and pregnancy complications increase dramatically. Older women are disproportionately represented among the population treated for infertility, particularly older primiparae, and a growing proportion of births are the result of assisted reproductive technology, such as *in vitro* fertilization (IVF) or intracytoplasmic sperm injection (ICSI) [5]. Researchers have sought to understand the underlying mechanisms behind the decline in fertility and embryo quality with advancing maternal age. The selection of embryos for transfer is one of the most crucial decisions during an IVF/ICSI procedure, as successful implantation and the birth of a healthy child depend upon the accuracy of the aforementioned selection. The basic, non-invasive method of identifying the embryo with the best prognosis for implantation is the assessment of embryonic morphology using the Gardner scoring system, which is based on the degree of embryonic expansion, as well as the morphology of the inner cell mass (ICM) and the trophectoderm (TE) [6]. Besides, morphological defects during early embryo development are also taken into account, such as cellular fragmentation, blastomere multinucleation, asymmetry of blastomeres, irregular shape, and vacuoles [7–10]. The assessment is normally carried out by an experienced embryologist, whose decision may be supported by time-lapse systems which provide additional, morphokinetic data [11]. Noteworthy, for over a decade, PGT-A has become the most important tool for the selection of healthy embryos for transfer [12]. Despite the advantages of using PGT-A in the IVF procedure, many untested embryos are still transferred due to the lack of indications for PGT-A, the young age of the patient, the patient’s decision to reduce costs, or the use of cryopreserved untested embryos. Moreover, PGT-A is not authorized in all countries. Therefore, numerous attempts have been made to predict the success of implantation and the birth of a healthy child on the basis of various morphological and morphometric parameters of the blastocyst, such as inner cell mass size and shape, the ratio of the size of ICM to the diameter of the blastocyst, and measurements of blastocyst expansion, especially when generated form advanced age patients [13–16]. Blastocoel expansion is also an important predictor of embryo transfer outcome. Some authors have reported that the degree of blastocoele expansion is positively correlated with implantation rate and that spontaneously hatching blastocysts (Bl. 5) have a better potential than expanded blastocysts (Bl. 4) to implant and establish a pregnancy [17–19]. Additionally, correlations between the expansion rate of blastocysts and their ploidy were found [20]. Blastocyst diameter and expansion rate are difficult parameters to analyze due to their dynamic nature, but in recent years, knowledge about the process of blastocyst expansion has been improved thanks to time-lapse analysis [20–22]. In embryological practice, when selecting a blastocyst for ET, apart from its morphology and degree of expansion, we also take into account its ability to hatch and often notice expanding blastocysts with a large diameter that do not hatch or smaller ones that are hatching. In such a situation, there is a dilemma of which blastocyst should be selected for transfer. Therefore, the aim of this study was to elucidate if the blastocyst size can be a reliable parameter for IVF clinics that cannot perform preimplantation tests for ploidy. Moreover, we intend to shed light on blastocyst’s ability to expand or hatch with regard to maternal age.

## MATERIALS AND METHODS

This was a retrospective study of 502 couples who underwent ICSI cycles at a Krakovi Clinic in Kraków (Poland) from 2021–2024. Institutional ethics committee approval (KBKA/06/O/2024) was obtained.

### Inclusion criteria

Women aged 26–45 years who underwent ICSI, PGT-A, and single embryo transfer of a euploid blastocyst were included in this retrospective study. Only expanded (Bl. 4) or hatching (Bl. 5) blastocysts of excellent, good, or medium quality were included in the study. Table 1, shows the general characteristics of the patients and blastocysts.

**Table 1:** General characteristics of the patients and blastocysts.

	**Maternal age**		**Total**
	**≤35 Y**	**>35 Y**	
Number of patients	211	291	502
Age (years), mean ± SD	32.5 ± 2	38.7 ± 3	35.3 ± 4
BMI (kg/m^2^), mean ± SD	22.6 ± 4	22.8 ± 3	22.7 ± 3
AMH (ng/ml), mean ± SD	3.22 ± 3.2	2.1 ± 2	2.7 ± 2.2
Indications for IVF			
Female factor, %	53%	51%	52%
Male factors, %	19%	18%	19%
Combined, %	28%	31%	29%
No of blastocysts, *n*	654	496	1150
No of blastocysts/patient, mean ± SD	3.1 ± 2	1.7 ± 1	2.3 ± 1.3
BL. 4 - Expanded blastocysts, *n* (%)	307/654 (47%)^a^	328/496 (66%)^c^	635/1150 (55%)
BL. 5 - Hatching blastocysts, *n* (%)	347/654 (53%)^a^	168/496 (34%)^c^	515/1150 (45%)
Total euploid rate, *n* (%)	405/654 (62%)^a^	188/496 (38%)^c^	563/1150 (49%)
Euploid rate BL. 4, *n* (%)	184/307 (60%)^a^	105/328 (32%)^c^	289/635 (45%)
Euploid rate, BL. 5, *n* (%)	222/347 (64%)^a^	67/164 (41%)^c^	289/515 (56%)
Diameter (µm), mean ± SD, range	192 ± 11 (155–225)	188 ± 6 (155–219)	190 ± 11 (155–225)
Diameter BL. 4 (µm), mean ± SD, range	186 ± 15 (155–225)	183 ± 10 (155–221)	185 ± 12 (155–225)
Diameter BL. 5 (µm), mean ± SD, range	196 ± 11 (158–223)	193 ± 7 (165–210)	195 ± 10 (158–223)
Small diameter BL. 4 (<185 µm), *n* (%)	172/307 (56%)^a^	151/328 (46%)^b^	323/635 (51%)
Small diameter BL. 5 (<195 µm), *n* (%)	177/347 (51%)^a^	64/168 (38%)^b^	221/515 (43%)
Large diameter BL. 4 (≥185 µm), *n* (%)	135/307 (44%)^a^	177/328 (54%)^b^	312/635 (49%)
Large diameter BL. 5 (≥195 µm), *n* (%)	170/347 (49%)^a^	104/168 (62%)^b^	274/515 (53%)

Abbreviations: AMH: Anti-Mullerian hormone; BMI: the body-mass index is the weight in kilograms divided by the square of the height in meters. ^a:b^values with different superscripts within the same rows differ significantly (*p* < 0.05), ^a:c^differ highly significantly (*p* < 0.001).

### Study design

**Figure 1:**
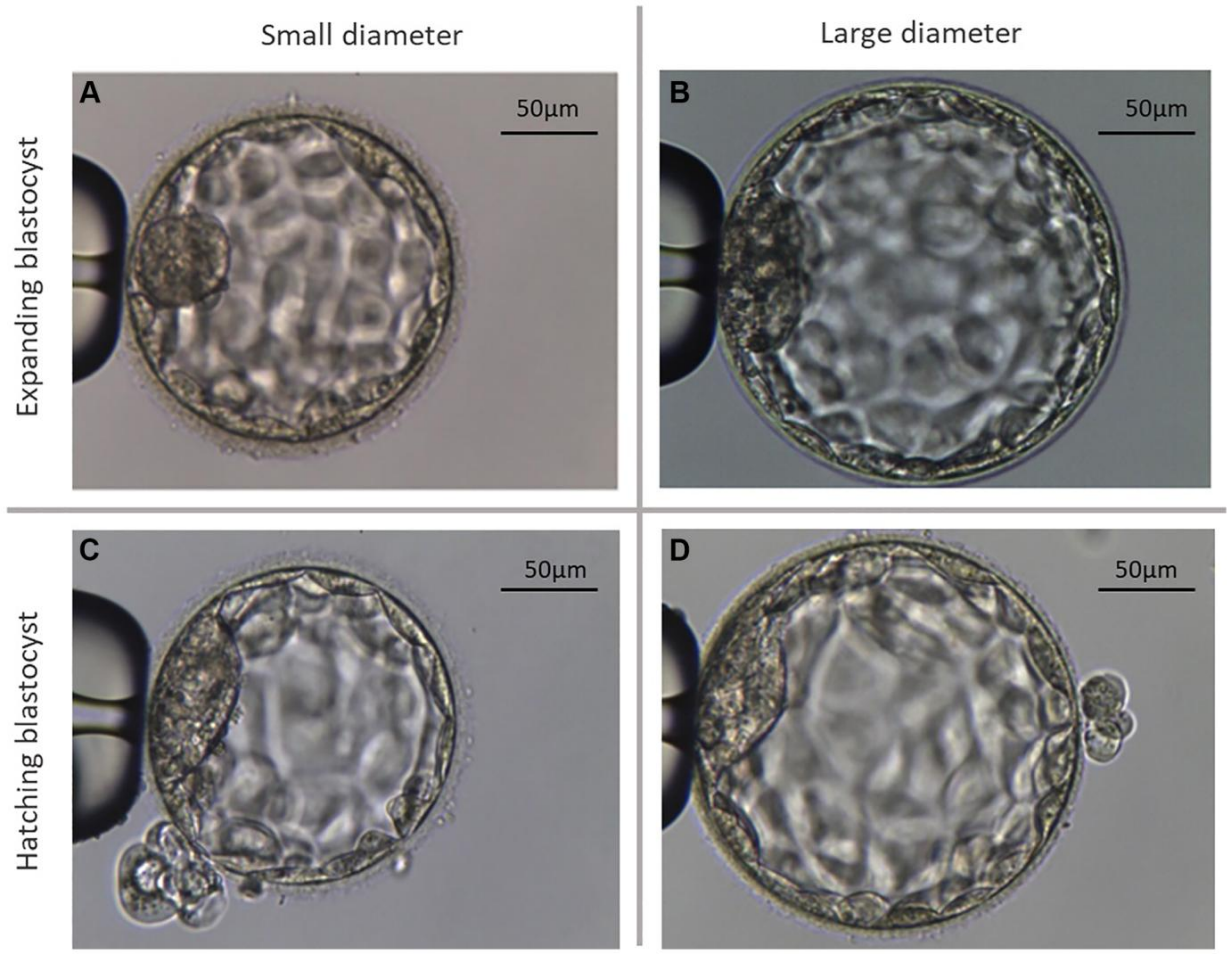
**Representative microscopic photographs of the two different types of analyzed blastocysts.** (**A**) Shows an exemplary photograph of a large expanding blastocyst (group Bl. 4; ≤185 µm). (**B**) Shows an exemplary photograph of a large expanding blastocyst (group Bl. 4; >185 µm). (**C**) Shows a small hatching blastocyst (group Bl. 5; ≤195 µm). (**D**) Shows a large hatching blastocyst (group Bl. 5; >195 µm).

**Figure 2:**
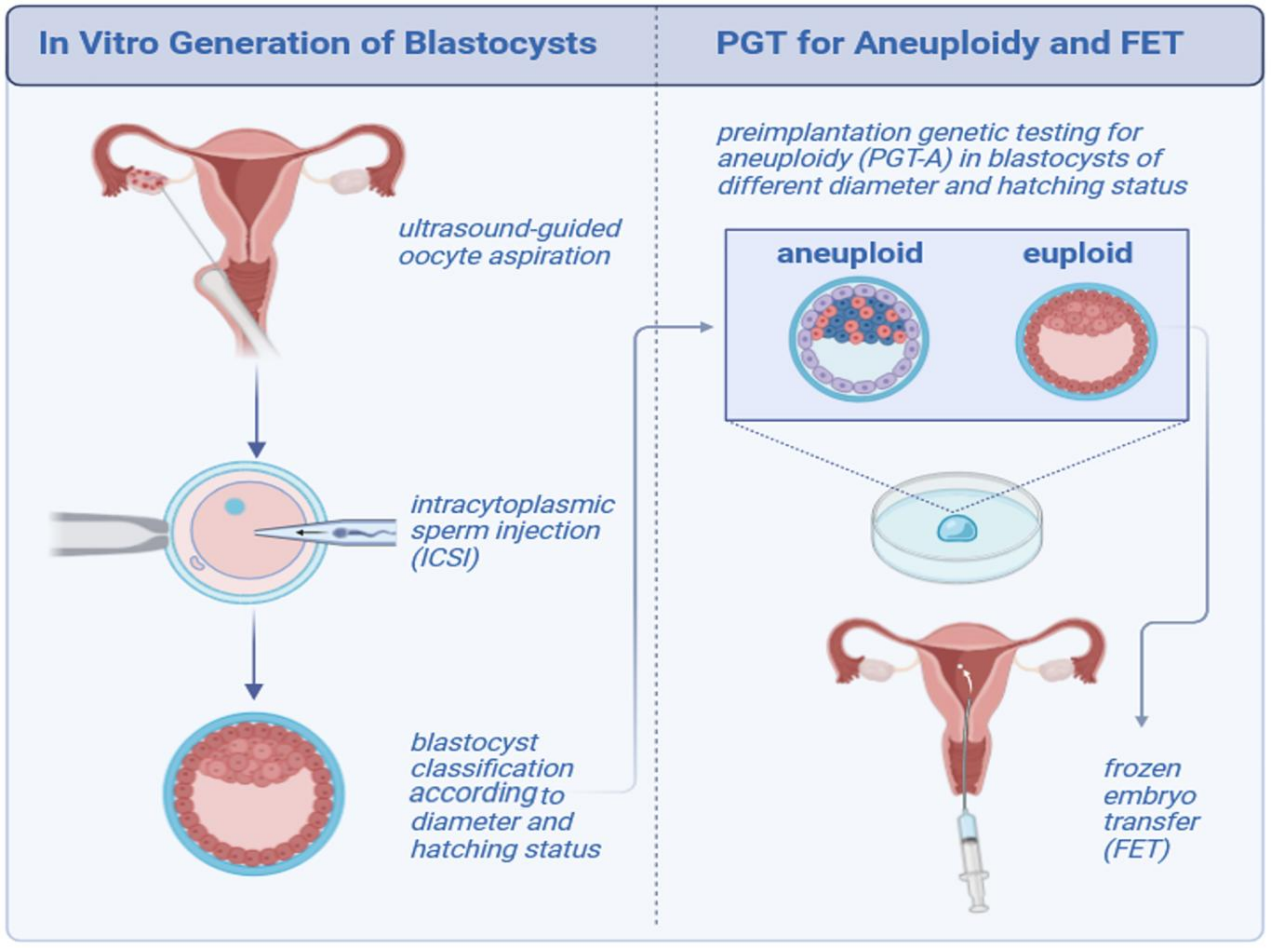
Scheme showing the study design.

In the first step of the study, as depicted in the scheme below, the diameter of 1150 blastocysts was analyzed to classify them as “small” or “large” blastocysts, and then in the second step to select “large unhatched” and “small hatched” blastocysts, respectively (Figure 1). As a third step, the PGT-A results were analyzed between these two groups of blastocysts. Finally, the outcome of FET for 201 euploid large unhatched and small hatched blastocysts was analyzed (Figure 2).

### Clinical protocols

Patients were treated using either the long agonist protocol or the short antagonist protocol, as will be described below. The agonist and antagonist protocols were continued up to and including the day of human chorionic gonadotropin (hCG; Eutrig, IBSA Farmaceutici) administration, which was when the leading follicle reached a diameter of 18 mm or more and at least three follicles reached a diameter of 17 mm or more. Recombinant Follicle Stimulating Hormone (rFSH; Folitropin alfa, Bemfola, Gedeon Richter) was then stopped, and a single s.c. bolus of 10,000 IU hCG (Eutrig 5000 IU, IBSA Farmaceutici) or 6,500 IU of recombinant human chorionic gonadotropin (rhCG/Ovitrelle 250 ug, Merck Serono) was administered 36 h before the planned time of oocyte retrieval. All follicles 12 mm or larger were aspirated. In case of a potential risk of ovarian hyperstimulation syndrome (OHSS) in an antagonist cycle, the trigger to induce ovulation was a single s.c. bolus of triptorelin 2 mg, and a freeze-all policy was applied.

### Antagonist protocol

A gonadotrophin realising hormone (GnRH) antagonist, Cetrorelix (Cetrotide, 0.25 mg/d, Merck Serono) or Ganirelix (Orgalutran 0.25 mg/d, Organon), was administered sc, commencing when the largest follicle reached a diameter of 14 mm. rFSH/human menopausal gonadotrophin (hMG; Menopur, Ferring Pharmaceuticals) was initiated on day 2–4 of the cycle.

### Long agonist protocol

Starting one week before the expected menses (cycle day 18–23), patients received sc the GnRH agonist, triptorelin (Decapeptyl, Gonapeptyl Daily, 0.1 mg/d, Ferring GmbH). After successful pituitary downregulation (when the serum estradiol (E2) levels were <40 pg/mL), ovarian stimulation was commenced with a fixed daily dose of 150–300 IU rFSH sc with or without an additional 75–150 IU hMG.

### Frozen embryo transfer (FET)

Treatment with oral E2 (17beta-estradiol 2 mg, Estrofem, Novo Nordisk, Denmark) was started on the first, second or third day of the cycle to prime the endometrium and suppress spontaneous follicle growth. Oral estradiol was administered in an incremental fashion: 2 mg/day during days 1–7, 4 mg/day during days 8–12, and 6 mg/day during days 13 to embryo transfer. Usually, after 12–14 days of E2 administration, a vaginal ultrasound examination was performed for endometrial thickness measurement and to confirm the absence of a leading follicle. When the endometrial thickness was >7 mm, progesterone (P4, Progesteronum 400 mg, Cyclogest, Gedeon Richter) supplementation was commenced, and the timing of FET was scheduled accordingly. For true natural cycle (t-NC), transvaginal sonography (TVS) was performed on day 2 or 3 of menses to rule out any cyst or *corpus luteum* remaining from the previous cycle. Cycle cancellation was usually undertaken in cycles with serum P4 >1.5 ng/ml on day 2 or 3 of menses. Transvaginal ultrasonographic monitoring was usually started on days 8–10, and endocrine monitoring, measuring serum E2, luteinizing hormone (LH) and P4, was performed when the leading follicle attained a mean diameter of approximately 15 mm in diameter. Following frequent endocrine and ultrasonographic monitoring, the day of ovulation was precisely documented on alternate days or daily to schedule the timing of FET.

### Laboratory protocols

Oocyte-cumulus complexes (COCs) were identified using a stereoscopic microscope and then washed and incubated (approx. 3 h) in the washing medium (Gynemed, Germany) under a 6.0% CO_2_, 5.0% O_2_ atmosphere. After incubation, oocytes were denuded using hyaluronidase and mechanical pipetting. Only oocytes in metaphase II with a first polar body were used for further procedures. For ICSI, fresh semen obtained by masturbation and analyzed according to WHO guidelines (2021) was used. A sperm sample was prepared for ICSI by the density gradient method (Gynemed, Germany). ICSI was performed using a Nikon Eclipse CS100 microscope and an RI Integra 3 micromanipulator (Research Instruments, Germany), following the standard technique. Embryos were *in vitro* cultured in SAGE^®^ medium (Origio, Denmark) in an atmosphere of 6.0% CO_2_, 5.0% O_2_, and balanced N_2_ at 37°C. Blastocysts were graded according to the Gardner scoring criteria (1) based on the degree of expansion, as well as ICM and TE morphology. Only “excellent” (Bl. 4AA, 5AA), “good” (Bl. 4AB, 4BA, 5AB, 5BA), and “medium” quality (Bl. 4 BB, 5BB) blastocysts were included in the study. Blastocysts were biopsied 120–124 h after ICSI, using the same micromanipulator and microscope used for ICSI. The zona pellucida was perforated using an Octax^®^ laser (Vitrolife, Sweden) for 250 μsec. The biopsied TE cells were washed with Dulbecos phosphate-buffered saline (D-PBS) and placed in 0.2 mL polymerase chain reaction (PCR) tubes for referral to Igenomix Inc. (Spain) and analysed by next-generation sequencing (NGS). Following the biopsy, blastocysts were incubated for 1.5 h in Sage^®^ medium and then vitrified. According to the manufacturer’s protocol, blastocysts were vitrified using Kitazato^®^ media and the Cryotop device (Kitazato, Japan). Blastocysts were warmed in Kitazato media for a minimum of 1.5 h before transfer and then placed in EmbryoGlue^®^ medium (Vitrolife, Sweden) or in Sage^®^ medium. More than 95% of transfers were performed in Embryo Glue^®^ medium. Assisted hatching was not performed since large unhatched blastocysts had a very thin zona pellucida and small ones began to hatch spontaneously.

### Blastocyst measurements and classification

Blastocyst measurements were made from images using MultiScan^®^ software. Blastocysts were graded twice, for the first time at 120 ± 1 h after ICSI, to grade the degree of expansion and qualify for biopsy. Two measurements were made for each blastocyst, including the zona pellucida and the average value of the diameter was determined. All activities related to blastocyst measurement were always performed by two embryologists. The average diameter for hatching blastocysts was 195 ± 12 µm, and for expanding blastocysts was 185 ± 10 µm. On this basis, Bl. 5 with a diameter <195 µm was classified as “small Bl 5” and Bl 5 ≥195 as “large Bl. 5”. Expanding blastocysts with a diameter <185 µm were classified as “small Bl 4” and Bl 4 ≥185 as “large Bl. 4”.

### Interpretation of PGT-A results

The diagnosis algorithm generates a graph representing the chromosomal copy number variation (CNV) of the sample analysed when compared to the reference bioinformatics baseline generated from multiple normal samples. An embryo is considered euploid when the graph shows no “threshold” deviations from the reference bioinformatics baseline for any of the 24 chromosomes assessed. “Threshold” embryos with less than 30% full chromosome aneuploidy and less than 50% segmental and sex chromosome aneuploidy in the biopsy are reported as euploid. An embryo is considered aneuploid when an aneuploidy or partial aneuploidy is detected as a result of a “Threshold” deviation from the reference bioinformatics baseline with points shifting upwards for a gain (trisomy) and downwards for a loss (monosomy). Partial aneuploidies are specified with chromosome number, arm (p,q), cytoband and fragment size in megabases (Mb). “Threshold”- embryos with more than 30% full chromosome and/or 50% segmental and sex chromosome aneuploidy in the biopsy are reported as aneuploid (according to Igenomix Laboratory, Spain).

### Statistical analysis

Non-parametric data, such as differences in the percentage values between groups, were assessed by the Chi-Square test since we did not assume a specific distribution of the variables measured on nominal and ordinal scales. Parametric data were expressed as means ± SD and compared by two-way ANOVA to draw conclusions about the populations which the data were generated from. Differences were considered significant when the *P*-value was ≤ 0.05. The statistical analysis was performed using PQStat 1.6.2 (PQStat Soft, Poznan, Poland).

Chi-Square test formula:


$$x_{c}^{2}=\frac{\Sigma {\left(O_{i}-E_{i}\right)}^{2}}{E_{i}}$$

Where: *c* = Degrees of freedom; *O* = Observed Value; *E* = Expected Value.

## RESULTS

**Figure 3:**
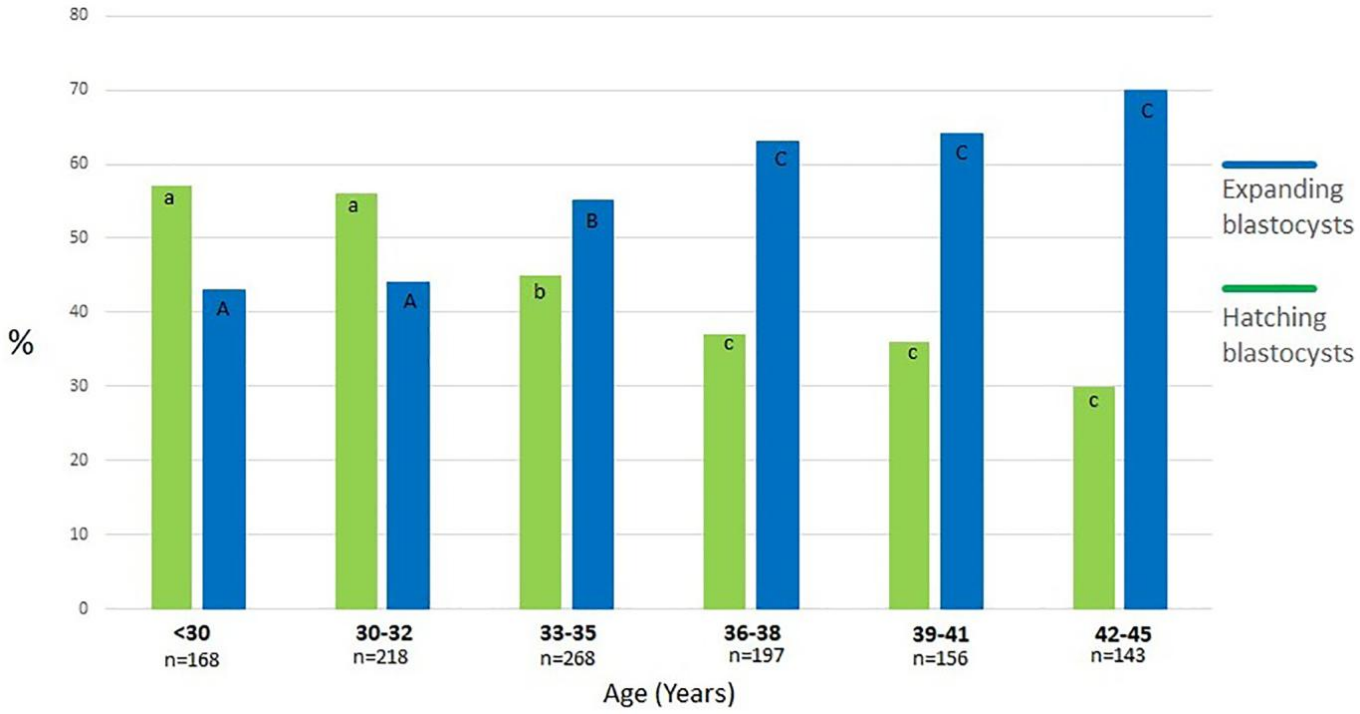
**Percentage of expanding and hatching blastocysts in different age groups.** a:b, A:B, b:c, B:C, a:A, b:B - values with different superscripts within bars differ significantly (*p* < 0.05), a:c, A:C, c:C - differ highly significantly (*p* < 0.001).

**Figure 4:**
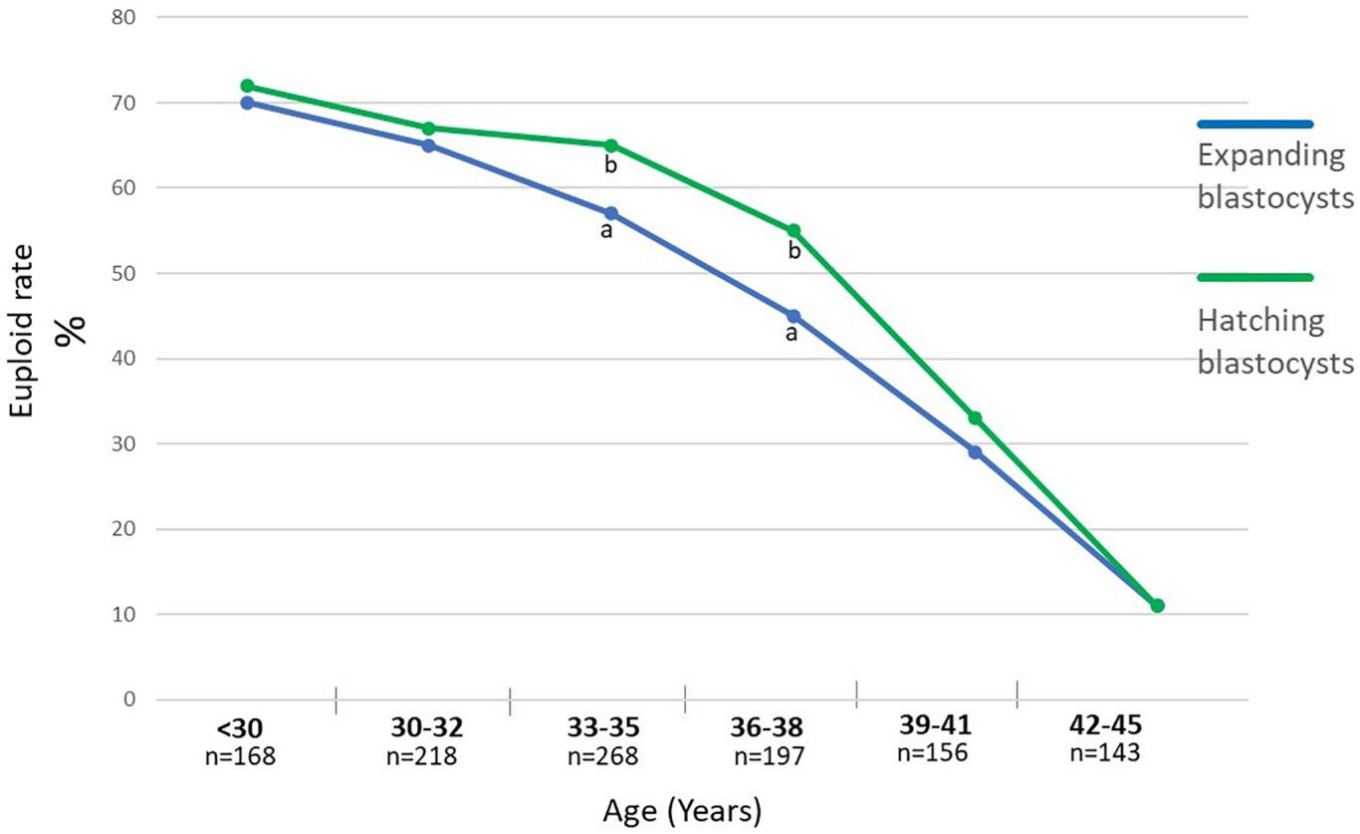
**Percentage of euploid blastocysts with regard to their stadium of development, meaning expanding and hatching blastocysts with regard to the maternal age.** a:b - values with different superscripts within points differ significantly (*p* < 0.05).

**Figure 5:**
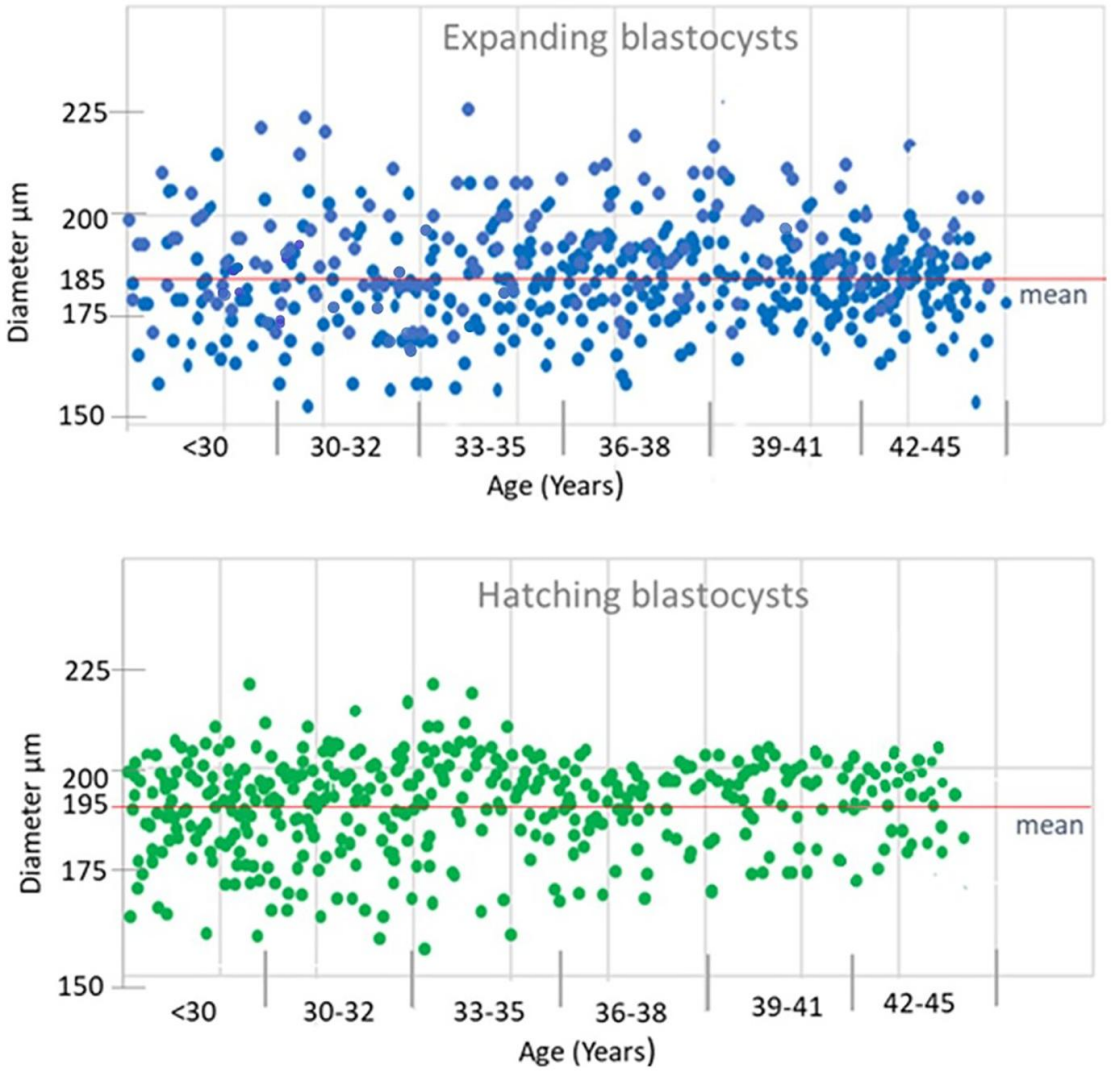
Size (diameter) distribution at expanding and hatching blastocysts in different age groups.

In the first phase of the study, 1150 blastocysts were measured; 635 (55%) expanded blastocysts (Bl. 4) and 515 (45%) hatching blastocysts. In patients with advanced maternal age, we observed a significantly lower percentage of hatching blastocysts compared to younger women (34% (≤35 Y) vs. 53% (>35 Y), *p* < 0.001) (Table 1, Figure 3). Moreover, Table 1 shows the results of blastocyst morphometry depending on maternal age and ability to hatch. The greater average diameter (195 ± 10 µm) was recorded in the hatching blastocyst group with a range of 158–223 µm in relation to the expanding ones (185 ± 12 µm) with a range of 155–225 µm. Among the expanding blastocysts, 49% (*n* = 312) were classified as “large Bl. 4”, meaning a diameter of ≥185 µm. In patients >35 years of age, there were larger Bl. 4 compared to younger patients (54% vs. 44%, *p* < 0.05). Among the hatching blastocysts, 43% (*n* = 221) were classified as “small Bl. 5” (≤195 µm). Significantly smaller Bl. 5 were noticed in the group of younger patients (51% vs. 38%, *p* < 0.05). The PGT-A testing of all 1150 blastocysts revealed that 563 (49%) were euploid. Figure 4 shows the percentage of euploid blastocysts with regard to their developmental stage and maternal age. In young patients (≤34 years of age) and in those with advanced maternal age (>38 years of age), no significant differences regarding euploidy were detected between the Bl. 4 and Bl. 5 groups. However, in patients aged 34–38 years, we detected 10% more euploid blastocysts in the hatching group than the expanding ones (*p* < 0.05). Figure 5 presents the size (diameter) distribution of expanding and hatching blastocysts in different age groups. Interestingly, in both the Bl 4 and Bl 5 blastocyst groups, a greater variation in embryo diameter was observed in younger patients, with a standard deviation of 15 vs. 10 for Bl 4 and a standard deviation of 11 vs. 7 for the Bl 5 group.

**Figure 6:**
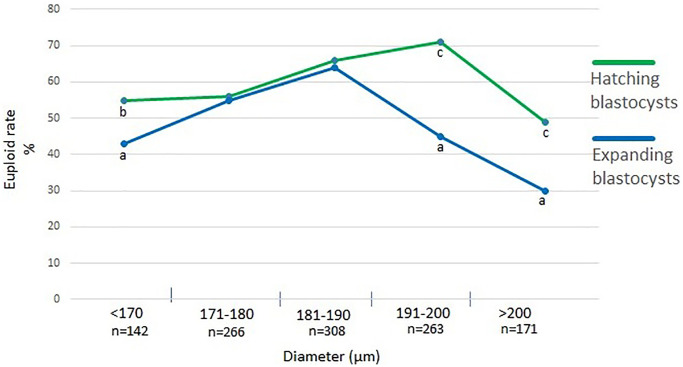
**Percentage of euploid blastocysts with regard to their diameter.** a:b - values with different superscripts within points differ significantly (*p* < 0.05), a:c - differ highly significantly (*p* < 0.001).

Figure 6 shows a percentage of euploid blastocysts with regard to their diameter. There is no difference in euploidy between Bl. 4 and Bl. 5 blastocysts with diameters of 170–190 µm. Noteworthy, those blastocysts with diameters either <170 µm or >190 µm, the hatching blastocysts have a higher euploid rate (<170 µm (*p* < 0.05), >190 µm (*p* < 0.001).

Then, the PGT-A results of blastocysts that started hatching at a small diameter and those that did not start hatching despite reaching a large diameter were analyzed in patients aged ≤35 years and >35 years (Table 2). In both age groups, the small hatching blastocysts (Bl. 5) group had a significant (*p* < 0.05) higher percentage of euploid embryos compared to large (Bl. 4), (73% vs. 58% in group ≤35 Y, 51% vs. 38% in group >35 Y). In the next stage, the FET results of 201 euploid blastocysts classified in the first stage as small hatching (*n* = 99) and large expanding (*n* = 102) were analyzed (Table 2). The implantation rate and pregnancy losses were similar between large and small euploid blastocysts, and no significant differences due to maternal age were recorded.

**Table 2:** Chromosomal status and implantation potential of blastocysts with regard to their diameter and ability to hatch.

**Maternal age (years)**	**Blastocyst size (diameter, µm)**	**Euploid rate *n* (%)**	**FET of euploid blastocyst**	
			**Implantation rate *n* (%)**	**Ongoing pregnancy *n* (%)**
**≤35 Y**	Large Bl. 4 (≥185 µm)	78/135 (58%)^a^	38/50 (76%)	36/50 (72%)
	Small Bl. 5 (<190 µm)	129/177 (73%)^b^	36/51 (71%)	35/51 (70%)
	Total	207/312 (66%)	74/101 (73%)	71/101 (70%)
**>35 Y**	Large Bl. 4 (≥185 µm)	67/177 (38%)^A^	37/52 (71%)	32/52 (61%)
	Small Bl. 5 (<190 µm)	33/64 (51%)^B^	32/48 (69%)	29/48 (60%)
	Total	100/241 (41%)	69/100 (69%)	61/100 (61%)

^a:b, A:B^values with different superscripts within the same column differ significantly (*p* < 0.05), ^a:A, b:B^differ highly significantly (*p* < 0.001).

## DISCUSSION

There is no doubt that maternal aging can contribute to the increased rates of aneuploidy observed in blastocysts generated from women of advanced age who undergo *in vitro* fertilization. The impact of maternal age on embryo quality and the likelihood of aneuploidy is well described, too. Our study provided evidence that in patients aged 34–38 years, there were 10% more euploid blastocysts in the hatching group compared to the expanding ones, which was a significant difference (*p* < 0.05). Women of advanced age have a higher rate of aneuploidy in their embryos, which can negatively impact implantation, sustained pregnancy, and live birth rates. Additionally, the hatching process of the blastocyst, which is crucial for successful implantation, may be impaired in aneuploid embryos. Moreover, aneuploid embryos often exhibit abnormal cell division and chromosomal distribution, disrupting the hatching process [23]. This being said, researchers have explored various interventions to mitigate the effects of maternal aging on IVF outcomes. Consequently, numerous attempts have been made to identify morphological markers of embryos that will predict their health and implantation potential. It has been well recognized that embryo morphology based on the Gardner scale is a significant predictor of implantation rate [23–25] and that euploidy rates are increased in blastocysts with better morphology [26, 27]. However, Alfarawati et al. reported that while about 52% of the excellent blastocysts were aneuploid, about 30% of the poorer-grade blastocysts were euploid, demonstrating why morphology alone cannot be relied on to ensure the transfer of euploid embryos [14]. The relationship between the degree of expansion and the implantation potential and ploidy of embryos has been equally well described. A few authors reported high implantation, pregnancy, and live birth rates with the transfer of hatching/hatched blastocysts [18, 28, 29]. Huang et al. showed a significantly higher number of euploid embryos in the region of highest expansion without taking into account spontaneous hatching [21]. Also, it has been observed that some blastocysts fail to hatch despite the high degree of expansion and thinning of zona pellucida (ZP) [30]. In our study, we wanted to compare the ploidy and implantation potential of small-diameter blastocysts that start hatching and large-diameter blastocysts that have not started to hatch. When analyzing the ability to hatch based on the diameter, we found the highest percentage of euploid embryos (≤35 Y- 73%, >35 Y- 51%) in the group of blastocysts that started the hatching process with a small diameter. In contrast, large, expanded, good, and excellent-quality blastocysts that had not started hatching had significantly more chromosomal defects than small-hatching blastocysts (≤35 Y- 58%, >35 Y- 38%). Of the 1150 blastocysts that underwent PGT-A analysis in this study, 49% were aneuploid. This is a similar result compared to other studies, where the percentage of aneuploidies usually exceeds 50% [14, 31, 32]. Based on the study results, it appears that the degree of expansion alone, even in good and excellent quality blastocysts, is not suitable for selecting embryos for ET or predicting their ploidy. Achieving a pregnancy and a healthy baby relies not only on the embryo’s chromosomal status but also on its implantation potential and its ability to maintain a pregnancy. Some investigators have indicated that blastocyst expansion is an important predictor of embryo transfer outcome [17–19, 33, 34], and a meta-analysis of FET outcomes found that the degree of blastocoele expansion, rather than the grade of ICM/TE, significantly affected the likelihood of pregnancy [35]. Many significant functions in the process of blastocyst implantation occur around the blastocoele expansion stage. These functions involve hCG-mediated signaling, adhesion, invasion of the endometrium, and maternal recognition of pregnancy, which are essential for successful implantation and proper development of pregnancy [14, 36–38]. It seems logical that good-quality blastocysts with large diameters would have more trophectoderm cells, providing them with a greater implantation potential. On the other hand, some previous studies suggested that blastocysts vitrified at an earlier developmental stage and with smaller blastocoeles have a higher cryosurvival rate and higher implantation rate after thawing [39–41]. In our study, after FET of euploid embryos, the implantation rate and pregnancy losses were similar between large expanded and small hatching blastocysts. However, it should be remembered that only euploid embryos were transferred, and assisted hatching and collapse were performed before the biopsy, which could have improved the cryo-survival rate of large blastocysts.

A limitation of our research appears to be the clinical difficulties in interpreting PGT-A results due to the occurrence of mosaic embryos. In our study, we used a binary classification of embryos, where full euploid and low-level mosaic embryos are classified as euploid embryos and full aneuploid and high-level mosaic embryos are classified as aneuploid. In clinical practice, a simplified classification into euploid and aneuploid is often used because we do not know the true level of mosaicism. The level of mosaicism obtained from PGT-A depends on the number of cells in a specific sample and the quality of these cells (viability, apoptosis). Moreover, the presence of mosaicism in a TE biopsy may not reflect the chromosomal constitution of the whole embryo. According to the literature, low-medium mosaicism embryos have equivalent developmental potential as fully euploid ones, and no significant difference was observed between infants from euploid and mosaic blastocyst transfers [42–44]. On the other hand, other authors reported that the embryos classified as low-level mosaic embryos showed a significant reduction in implantation compared with euploid embryo transfers by 12% [45]. Thus, the discussion on reporting and transferring mosaic embryos is still relevant, and these procedures should be standardized.

## CONCLUSIONS

In conclusion, when selecting non-PGT-A tested embryos for ET/FET, a small hatching blastocyst is a better choice than a large expanded one, especially for advanced maternal-age patients for whom the risk of aneuploidy is higher. This might be related to the fact that the zona pellucida of aged oocytes tends to be harder, and therefore, the presumptive blastocysts expand but have difficulties hatching. Moreover, the increase of aneuploidy and the lower ability to hatch is also related to the fact that mammalian oocytes are in the ovary from the beginning of life and, consequently, exposed to numerous environmental and epigenetic insults. However, when transferring euploid embryos, there are no differences in the implantation potential between large expanded or small hatching blastocysts, which leads to the conclusion that aneuploidy correlates with decreasing hatching.
